# *SNAI2*/Slug promotes growth and invasion in human gliomas

**DOI:** 10.1186/1471-2407-10-301

**Published:** 2010-06-17

**Authors:** Hong Wei Yang, Lata G Menon, Peter M Black, Rona S Carroll, Mark D Johnson

**Affiliations:** 1 Department of Neurosurgery, Brigham and Women's Hospital and Harvard Medical School, 75 Francis Street, Boston, MA, USA 02115

## Abstract

**Background:**

Numerous factors that contribute to malignant glioma invasion have been identified, but the upstream genes coordinating this process are poorly known.

**Methods:**

To identify genes controlling glioma invasion, we used genome-wide mRNA expression profiles of primary human glioblastomas to develop an expression-based rank ordering of 30 transcription factors that have previously been implicated in the regulation of invasion and metastasis in cancer.

**Results:**

Using this approach, we identified the oncogenic transcriptional repressor, *SNAI2*/Slug, among the upper tenth percentile of invasion-related transcription factors overexpressed in glioblastomas. *SNAI2 *mRNA expression correlated with histologic grade and invasive phenotype in primary human glioma specimens, and was induced by EGF receptor activation in human glioblastoma cells. Overexpression of *SNAI2/*Slug increased glioblastoma cell proliferation and invasion *in vitro *and promoted angiogenesis and glioblastoma growth *in vivo*. Importantly, knockdown of endogenous *SNAI2*/Slug in glioblastoma cells decreased invasion and increased survival in a mouse intracranial human glioblastoma transplantation model.

**Conclusion:**

This genome-scale approach has thus identified *SNAI2*/Slug as a regulator of growth and invasion in human gliomas.

## Background

Malignant gliomas characteristically invade the surrounding brain, making them incurable by surgery alone. Glioma cell invasion depends upon multiple factors, including extra-cellular matrix (ECM) molecules, growth factors, and the activity of intracellular pathways regulating cell motility [1]. However, the upstream mechanisms that control glioma invasion are poorly known.

Recent studies have revealed the existence of several transcription factors that control genetic programs promoting metastasis and invasion in human cancer [2]. Among these is Slug, an oncogenic transcriptional repressor that acts as a master regulator of cell migration in many tissues [3]. Slug is the product of the *SNAI2 *gene and is overexpressed in numerous cancers, including leukemia [4,5], esophageal cancer [6], lung cancer [7], breast cancer [8,9], ovarian cancer [8,10], prostate cancer [11], and colorectal cancer [12]. Transgenic mice overexpressing Slug develop leukemias and mesenchymal tumors, demonstrating an oncogenic role for this protein [13]. In addition to its effects on migration and tumorigenesis, Slug inhibits p53-dependent apoptosis by antagonizing the trans-activation of *PUMA *by p53 [14].

Despite the evidence that Slug is involved in several types of peripheral cancers, a role for Slug in human nervous system tumors has not yet been identified. We show here that *SNAI2*/Slug is overexpressed in a subpopulation of glioblastomas in an EGF-dependent manner, and *SNAI2/*Slug mRNA expression correlates with increasing tumor grade and invasive phenotype in human gliomas. We also demonstrate that *SNAI2*/Slug promotes invasion and growth in human glioblastomas.

## Methods

### Cell lines and tumor samples of astrocytoma and glioblastoma multiforme

All studies were performed after written informed consent was obtained under the auspices of a human subjects institutional review board (IRB) protocol approved by the Partners Human Research Committee. Primary frozen tissue from 78 human glioma specimens (including 15 low grade astrocytomas, 15 low grade oligodendrogliomas, 10 low grade gangliogliomas, 7 anaplastic astrocytomas and 31 glioblastomas (GBMs) were obtained from the Brain Tumor Tissue Bank in the Department of Neurosurgery at Brigham and Women's Hospital. Four human glioblastoma cell lines (U87, U251, U343, and T98) were obtained from the American Tissue Type Culture Collection. The D566 human glioblastoma cell line was a gift from D. Bigner, Duke University. All cell lines were cultured in DMEM (Invitrogen, Carlsbad, CA) supplemented with 10% FBS, and were maintained in a 5% CO_2 _incubator at 37°C.

### mRNA expression profiling

Total RNA was isolated from 20 fresh frozen human glioma samples and from 7 non-tumor brain samples. In some experiments, RNA was isolated from U87 human glioblastoma cells after transduction with a *SNAI2*/Slug lentivirus or a control virus. The mRNA was reverse-transcribed to generate cDNA, which was then biotinylated and hybridized to Affymetrix HG-U133A expression arrays prior to scanning for quantitation. For data from primary glioma specimens, expression heatmaps were constructed using expression data from the non-tumor brain specimens as a reference. Statistical comparisons between histologic subgroups were performed using the t-test.

### Taqman Real-time PCR

Total RNA was extracted from cell lines with TRIzol (Invitrogen, Carlsbad, CA), according to the manufacturer's protocol. Randomly primed cDNA was prepared using 1 μg of total RNA from each sample and the AMV 1st Strand cDNA Synthesis Kit (Roche Applied Science, Indianapolis, IN). Six ng of each cDNA were then used for real-time PCR analysis in a final reaction volume of 20 μl. Probes for β-actin (Hs99999903_m1) and human *SNAI2*/Slug (Hs00161904_m1) were purchased from Applied Biosystems (Foster City, CA). Samples were analyzed in triplicate using the ABI 7300 software system (Applied Biosystems, Foster City, CA) with DDCt quantification. Statistical analysis was performed using the t-test.

### Lentivirus production and establishment of stable glioma lines

The full length human *SNAI2 *gene was cloned by RT-PCR from U87 cells using the forward primer 5'- CAC CAT GCC GCG CTC CTT CCT GGT C-3' and the reverse primer 5'-TCA GTG TAC ACA GCA GCC AGA-3', and was subsequently transferred into the pLenti6-IRES-EGFP vector. The correct *SNAI2 *sequence was confirmed by direct DNA sequencing. A lentiviral shRNA vector for Slug and an appropriate empty control vector were purchased from Open Biosystems (TRCN0000015389, Huntsville, AL). The lentiviral vectors were packaged in 293FT cells using the ViraPower Lentiviral Expression System (Invitrogen, Carlsbad, CA) according to the manufacturer's protocol.

Human U251 and U87 glioblastoma cells were transduced with the appropriate lentiviruses, and stable cell lines (U251-IRES-GFP and U251-Slug-IRES-GFP) were selected using 8 μg/ml blasticidin. U87-pLKO.1 and U87-shSlug stable cell lines were selected using puromycin.

### Growth and proliferation assays

To measure cell growth, 1 × 10^3 ^glioma cells were plated into 96 well culture plates in triplicate, and cell growth was determined from day 0 to day 6 using a tetrazolium salt-based colorimetric assay (Cell Counting Kit-8, Dojindo Molecular Technologies, Gaithersburg, MD) according to the manufacturer's protocol.

To measure cell proliferation, 1 × 10^3 ^human glioma cells were plated into 96 well culture plates in triplicate and cultured for 6 hours. The cells were then incubated in BrdU overnight at 37°C. BrdU incorporation into DNA was quantitated by ELISA assay (Roche Applied Science, Indianapolis, IN) according to the manufacturer's protocol.

### Western blots

Total protein was extracted using RIPA buffer supplemented with proteinase inhibitors. Protein extracts were then separated by gel electrophoresis using 4-20% SDS-PAGE - Tris-HCl gels (Bio-Rad, Hercules, CA). The protein was transferred to nitrocellulose membranes and detected using a specific anti-Slug antibody (G-18, Santa Cruz Biotechnologies, Santa Cruz, CA) at a 1:1000 dilution. After washing and incubation in the appropriate secondary antibody, immunoreactive bands were visualized using the enhanced chemiluminescence system.

### Immunohistochemistry

Animals containing subcutaneous tumors were anesthetized, and the subcutaneous tumors were then harvested, fixed with 4% paraformaldehyde at pH 7.4, immersed in 30% sucrose, embedded in OCT and cryostat-sectioned at 6 μm at -20°C. Hematoxylin and eosin (H&E) staining was performed, and immunohistochemistry was performed using the Vectastain Elite ABC kit (Vector Laboratories, Burlingame, CA). A specific anti-CD31 primary antibody (1:100; BD Biosciences PharMingen) was used to visualize blood vessels.

### In vitro cell migration and invasion assays

Cell migration was measured using the *in vitro *scratch wound healing assay [15] and transwell assay [16]. For the wound healing assay, U251-IRES-GFP and U251-Slug-IRES-GFP cells were cultured at 37°C in DMEM supplemented with 10% FBS until confluence was achieved. A cell-free area was then created by scratching the monolayer with a pipette tip, and the culture was photographed at time 0 and after 24 hours. The distance migrated from the edge of the scratch was measured using an automated imaging software program (SPOT, Diagnostic Instruments, Sterling Heights, MI).

For the transwell assay, 10 mm tissue culture inserts with polycarbonate membranes (8 μm pore, Nalge Nunc International, Rochester, NY) were used according to the manufacturer's protocol. Briefly, 1 × 10^5 ^U251-IRES-GFP or U251-Slug-IRES-GFP cells were suspended in DMEM supplemented with 1% FBS and plated into the 10 mm tissue culture inserts. DMEM supplemented with 10% FBS was used in the lower chamber as a chemoattractant, and the cells were allowed to migrate overnight. The cells that failed to migrate (located on the upper surface of the membrane) were removed using a cotton swab. The membranes were then stained with the 3 Step Stain Set (eosin-Y, azure A and methylene blue, Richard-Allen Scientific, Kalamazoo, MI), dried and mounted using Cytoseal 60. The cell number was determined by calculating the mean number of cells from five separate 40 × fields. The same protocol was used for the migration of U87-pLKO.1 and U87-shSlug cells, except that the migration time was decreased to 5 hours.

Invasion by glioblastoma cells *in vitro *was measured using the Matrigel invasion assay [17]. The BD BioCoat Matrigel invasion chamber (8 μm pore, BD Biosciences, San Jose, CA) was used according to the manufacturer's protocol. Briefly, 1 × 10^5 ^U251-IRES-GFP and U251-Slug-IRES-GFP cells were suspended in DMEM with 1% FBS and plated onto 10 mm tissue culture inserts. DMEM containing 10% FBS was placed into the lower chamber, and the cells were allowed to invade through the matrix overnight. All incubations were conducted at 37°C with 5% CO_2_. The cells that failed to migrate through the membrane were removed with a cotton swab, and the membranes were stained with the 3 Step Stain Set for blood smear (Richard-Allen Scientific, Kalamazoo, MI). The cell number was determined by taking the average from five separate 40 × fields. Statistical significance was determined using the t-test.

### Intracranial and subcutaneous tumor growth and survival assays

All animal experimental procedures were carried out in the animal facility at Brigham and Women's Hospital under the auspices of an approved protocol and in accordance with federal, local and institutional guidelines. For the subcutaneous tumor growth assay, approximately 5 × 10^5 ^U251-IRES-GFP or U251-Slug-IRES-GFP cells were subcutaneously injected into the flanks of 5-week-old nude mice. The tumor volumes were measured each week beginning with the second week after injection, and the tumors were allowed to grow for an additional 6 weeks. At the termination of the study, the mice were euthanized and the tumors were surgically removed and processed for histochemistry. A total of 4 mice were studied in each group. Tumor volume was calculated from the length (a) and width (b) using the formula V = 4/3π(ab^2^/2) [18]. Statistical significance was determined using the t-test.

For the intracranial orthotopic human glioma model, approximately 5 × 10^4 ^U87-pLKO.1 or U87-shSlug glioblastoma cells were injected intracranially into the frontal cortex of 5-week-old male nude mice. Six mice in the control group and 5 mice in the U87-shSlug group received intracranial human glioblastoma cell transplants. The animals were then followed until they showed signs of neurologic dysfunction or distress, at which time they were sacrificed. Kaplan-Meier survival analysis was then performed, and statistical significance was determined using the Logrank test.

## Results

### SNAI2/Slug expression correlates with grade and invasive phenotype in gliomas

**Figure 1:**
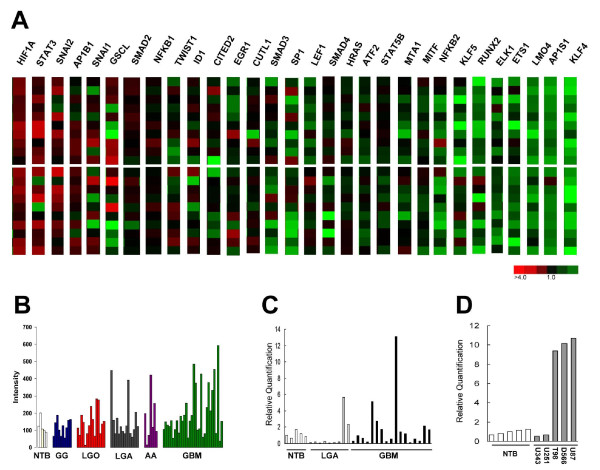
***SNAI2*/Slug mRNA is overexpressed in human gliomas and correlates with histologic grade and invasive phenotype**. ***A***) mRNA expression heatmaps for 30 migration/invasion-related transcription factors. Data shown was obtained from 20 primary human glioblastoma specimens (10 upper panel, 10 lower panel). Changes in glioblastoma gene expression are shown relative to mean expression values obtained from 7 non-tumor brain specimens. Heatmaps are ordered according to their degree of overexpression relative to non-tumor brain. ***B***) mRNA microarray data for *SNAI2 *expression in 79 human glioma specimens. Data shown was obtained using 5 non-tumor brain specimens (NTB), 10 supratentorial ganglioglioma (GG) specimens, 15 low grade oligodendroglioma specimens (LGO), 15 low grade astrocytoma specimens (LGA), 7 anaplastic astrocytoma (AA) specimens and 32 glioblastoma (GBM) specimens. Intensity data is plotted on the y axis. *SNAI2*/Slug mRNA expression was elevated in glioblastomas compared to low grade astrocytomas (*P *< 0.006, t-test). ***C***) Real-time PCR analysis of *SNAI2*/Slug mRNA expression in non-tumor brain (NB), low grade astrocytoma (LGA) and glioblastoma (GBM) specimens. ***D***) Relative quantification of *SNAI2*/Slu*g *mRNA expression using Real-time PCR in several human glioblastoma cell lines (U343, U251, T98, D566 and U87). Data from non-tumor brain specimens is shown for comparison.

To identify upstream determinants of invasiveness in human gliomas, we performed a screen of 30 transcription factors implicated in the regulation of invasion and metastasis in other cancers. These factors were selected after a search of the Pubmed database using the search terms transcription factor and metastasis, invasion or migration. Expression of the mRNA for these transcription factors was then examined in a panel of 20 human glioblastomas using mRNA microarray analysis, and the transcription factors were rank ordered based upon their degree of overexpression when compared to non-tumor brain. Using this approach, we identified *HIF1A, STAT3 *and *SNAI2 *(Slug) as the three most overexpressed migration-related transcription factors in human glioblastomas when compared to non-tumor brain (Figure 1A). Previous reports have indicated key roles for *HIF1A *and *STAT3 *in glioblastoma aggressiveness [17,19,20], confirming the validity of this rank-order approach for identifying upstream transcription factors regulating the invasive phenotype of human gliomas. However, a role for *SNAI2/*Slug in human gliomas has not been reported previously. The frequency and extent of *SNAI2*/Slug mRNA overexpression exceeded that of 26 of the 30 invasion/migration-related transcription factors examined, several of which have been implicated specifically in glioma invasion. Among these were *AP1, NFKB1*, and *TWIST1 *[21,22].

Different histologic subtypes of glioma show a differential tendency to invade the surrounding brain. Although gangliogliomas can infiltrate the surrounding brain parenchyma, they are often relatively well circumscribed, so that they can sometimes be cured by surgical resection [23]. In contrast, low grade diffuse fibrillary astrocytomas are generally more invasive than gangliogliomas, while low grade oligodendrogliomas display an intermediate invasive phenotype. In addition, glioma invasion correlates with tumor grade, with malignant gliomas (e.g. glioblastomas) being more invasive than low grade gliomas. To determine whether *SNAI2*/Slug expression correlates with these parameters, we examined *SNAI2*/Slug mRNA expression in 78 human gliomas of different histologic subtypes and grades (Figure 1B). Mean *SNAI2*/Slug mRNA expression was low in non-tumor brain (112 ± 42) and in gangliogliomas (119 ± 52), which have low invasive potential. Mean *SNAI2*/Slug mRNA levels were higher in low grade oligodendrogliomas (143 ± 78) and in low grade diffuse fibrillary astrocytomas (158 ± 120). Importantly, *SNAI2*/Slug mRNA expression was significantly higher in glioblastomas than in low grade diffuse astrocytomas or in non-tumor brain (*P *< 0.006, t-test). We confirmed overexpression of *SNAI2*/Slug mRNA by real-time PCR in 9 low grade astrocytomas (LGA) and 17 glioblastomas (GBMs). Elevated expression of *SNAI2*/Slug mRNA when compared to non-tumor brain (NB) was observed in 2 of 9 (22%) low grade astrocytomas and 6 of 17 (35%) glioblastomas (Figure 1C).

We also examined *SNAI2*/Slug mRNA expression in 5 human glioma cell lines. *SNAI2*/Slug mRNA expression was increased more than 9 fold in U87, D566 and T98 human glioma cell lines when compared to non-tumor brain tissues, while the U251 and U343 glioma cell lines showed *SNAI2*/Slug expression levels similar to those observed in non-tumor brain (Figure 1D).

**Figure 2:**
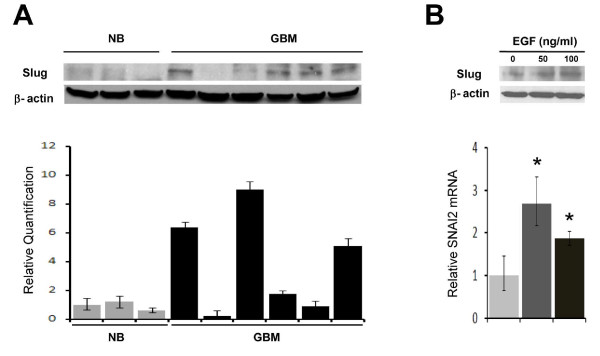
**Slug is overexpressed in glioblastoma and induced by EGF**. ***A) upper panel ***Western blot analysis of protein isolates derived from 6 human glioblastoma (GBM) and 3 human non-tumor brain (NB) specimens. Blots were stained using a specific anti-Slug antibody. β-actin was used as an internal reference for normalization. The Western blot analysis showed low or absent Slug protein expression in all three non-tumor brain specimens and increased Slug expression in five of six GBM specimens. *lower panel *mRNA was isolated from the same tumor specimens used in the upper panel and quantitative Real-time PCR for *SNAI2 *mRNA was performed as described. Data shown are the mean of three replicates. ***B) **upper panel *Slug protein was isolated from U251 human GBM cells after exposure to EGF (50 or 100 ng/ml) for 4 hours and assayed by Western blot. β-actin was used as an internal reference for normalization. *lower panel *Real-time PCR analysis of *SNAI2 *mRNA from the same samples displayed in the upper panel. Data shown are mean ± SEM of three replicates.

We next used Western blot analysis to determine Slug protein expression in primary human glioblastoma specimens. Slug protein was undetectable in 5 human non-tumor brain specimens. In contrast, Slug protein expression was elevated in 5 of 13 (38%) human glioblastoma specimens examined (Figure 2A). Slug protein expression correlated closely with *SNAI2*/Slug mRNA levels (Figure 2A).

In epithelial cells, *SNAI2*/Slug expression can be increased by EGF receptor activation[24]. We observed a significant increase in *SNAI2 *mRNA and protein in U251 glioblastoma cells after EGF exposure (Figure 2B). Although Slug is also a downstream effector of the SCF/c-kit pathway[25], we failed to detect an increase in *SNAI2*/Slug mRNA after SCF exposure in U251 glioblastoma cells (data not shown). Taken together, these data indicate that *SNAI2*/Slug is overexpressed in a subpopulation of human gliomas, and its expression correlates with invasive phenotype and tumor grade.

### SNAI2/Slug promotes growth and proliferation in glioblastoma

**Figure 3:**
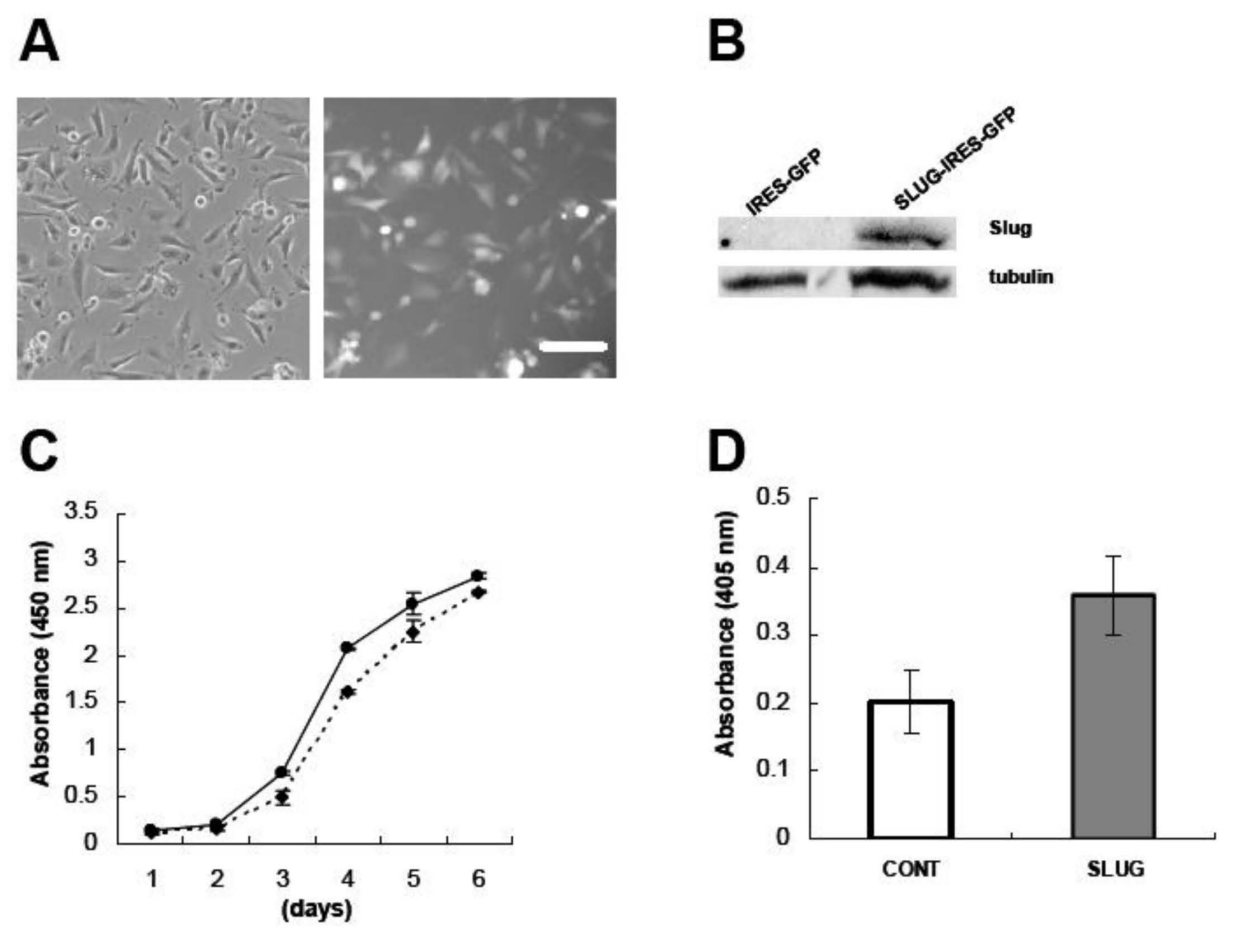
***SNAI2*/Slug overexpression increases glioblastoma growth and proliferation**. ***A***) Phase and fluorescence micrographs showing human U251 glioblastoma cells transduced with a Slug-IRES-EGFP lentivirus. ***B***) Western blot analysis demonstrating increased Slug protein expression in U251 glioblastoma cells transduced with the Slug-IRES-GFP lentivirus. ***C***) Colorimetric growth assay demonstrating growth curves for human U251 glioblastoma cells overexpressing a vector containing *SNAI2*/Slug (solid line) or an empty control vector (dashed line). Data shown are mean ± SEM. Slug-expressing glioblastoma cells grew at a significantly faster rate than control cells (*P *< 0.03, t-test). ***D***) BrdU incorporation into DNA for human U251 glioblastoma cells overexpressing an *SNAI2*/Slug vector or an empty control vector. Data shown are mean ± SEM. *SNAI2*/Slug-expressing glioblastoma cells synthesized new DNA at a significantly faster rate than control cells (asterisk indicates *P *< 0.00001, t-test).

To determine the consequences of *SNAI2/*Slug overexpression in human gliomas, we first generated an IRES-EGFP lentivirus containing the human *SNAI2 *gene. A lentivirus containing an empty IRES-EGFP vector was prepared as a control. Human U251 glioblastoma cells were then transduced with these lentiviruses and stable cell lines were produced (Figure 3A). Overexpression of Slug protein in U251 glioblastoma cells transduced with the *SNAI2*/Slug lentivirus was confirmed by Western blot (Figure 3B). *In vitro *growth assays indicated that glioblastoma cells overexpressing *SNAI2*/Slug grew significantly faster than control cells (Figure 3C, *P *< 0.03, t-test). To investigate this phenomenon further, we examined proliferation by measuring bromodeoxyuridine (BrdU) incorporation into DNA. Human U251 glioblastoma cells overexpressing Slug displayed a higher rate of DNA synthesis than did control cells (Figure 3D, *P *< 0.00001, t-test).

### Slug increases migration and invasion in human gliomas

**Figure 4:**
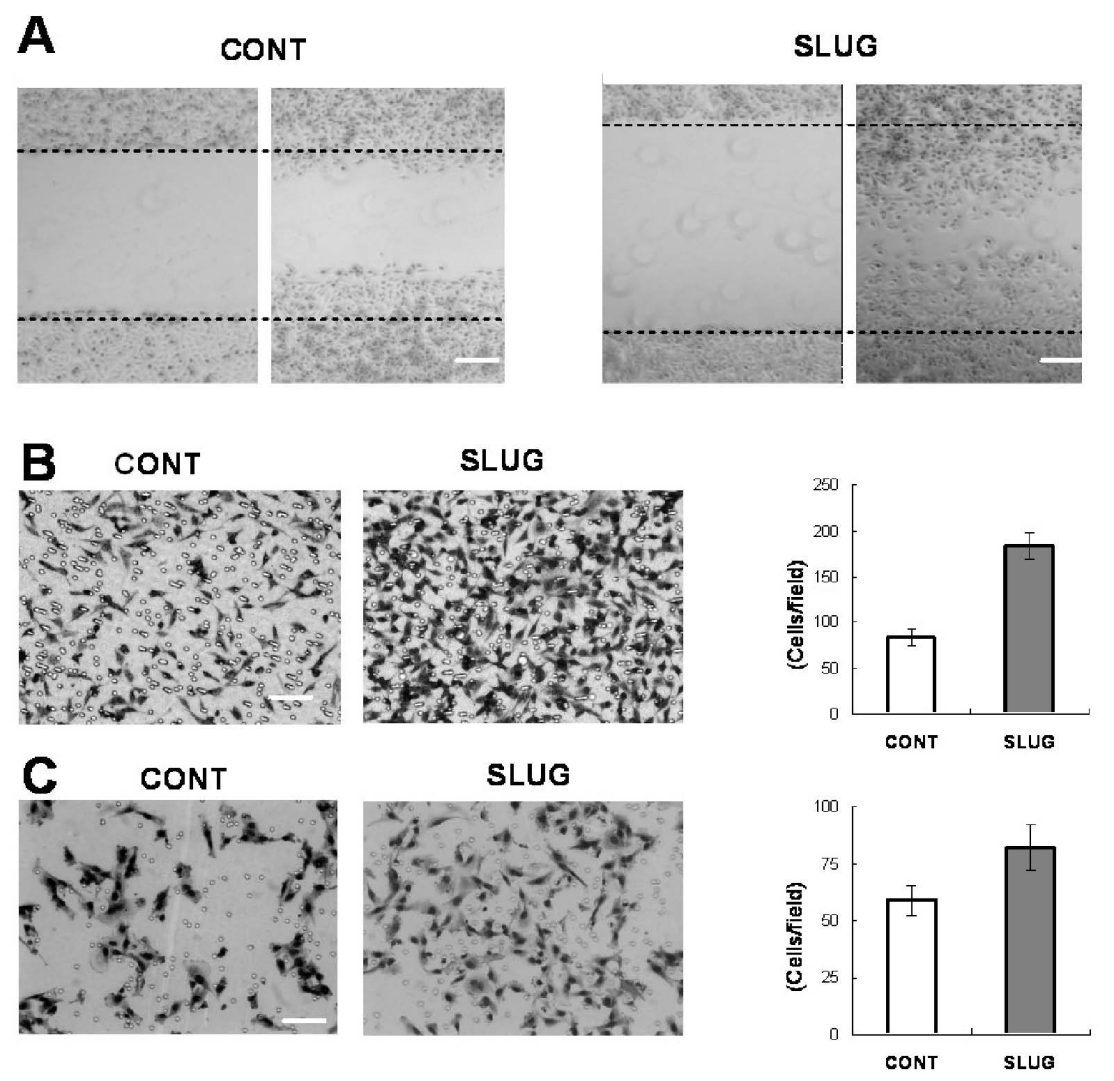
**Overexpression of *SNAI2*/Slug increases glioblastoma migration and invasion**. ***A***) Scratch wound healing assay for human U251 glioblastoma cells overexpressing a control vector or a *SNAI2*/Slug vector. Cells were photographed at 0 hours and 24 hours after scratch application. The dotted lines indicate the original edges of the scratch defect. ***B***) Transwell migration assay for human U251 glioblastoma cells overexpressing *SNAI2*/Slug or a control vector. 10% serum was used as a chemoattractant. The cells that migrated through the membrane were stained and counted under direct microscopy. Slug-expressing glioblastoma cells migrated faster than the control cells (asterisk indicates *P *< 0.00001, t-test). ***C***) 3-dimensional Matrigel assay for human U251 glioblastoma cells overexpressing a *SNAI2*/Slug vector or a control vector. After 24 hours, cells that invaded through the matrix were fixed, stained with H&E and counted under direct microscopy. Data shown are mean ± SEM. Glioblastoma cells overexpressing Slug showed greater invasiveness than did control cells (asterisk indicates *P *< 0.001, t-test).

Our analysis of *SNAI2/*Slug expression in primary glioma specimens suggested a relationship between *SNAI2*/Slug and invasive phenotype. To determine the basis for this relationship, we first performed an *in vitro *scratch wound healing assay using human U251 glioblastoma cells. Glioblastoma cells overexpressing *SNAI2*/Slug migrated approximately 62 μm from the edges and covered 63% of the scratch defect (Figure 4A), while control cells expressing the empty IRES-EGFP vector migrated only 39 μm from the edges and covered less than 39% of the scratch defect (Figure 4A, *P *< 0.00001, t-test).

We also examined the effect of *SNAI2/*Slug on chemotaxis in glioblastoma cells using a transwell migration assay and 10% serum as a chemoattractant (Figure 4B). Significantly more U251 glioblastoma cells overexpressing *SNAI2/*Slug migrated through the membrane than did control cells (184 +/- 15 cells/hpf versus 83 +/- 9 cells/hpf, *P *< 0.00001, t-test).

Finally, we utilized a three dimensional Matrigel invasion assay to determine the effect of *SNAI2*/Slug expression on glioma invasion (Figure 4C). We found that significantly more U251 glioblastoma cells overexpressing *SNAI2*/Slug passed through the matrix than did control cells (83 +/- 10 cells/hpf versus 59 +/- 7 cells/hpf, *P *< 0.001, t-test). Taken together, these results indicate that enforced overexpression of *SNAI2*/Slug increases migration and invasion in human glioblastoma cells.

**Figure 5:**
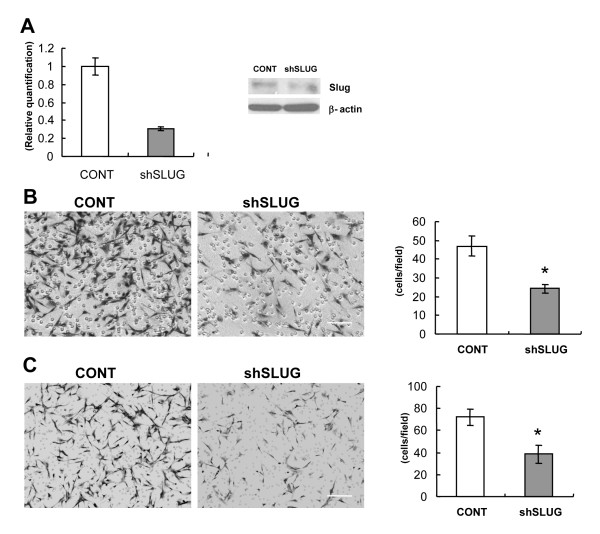
**Knockdown of endogenous *SNAI2*/Slug decreases glioblastoma migration and invasion**. ***A***) Human U87 glioblastoma cells were transduced to over express an shRNA directed against *SNAI2*/Slug (shSlug) or a control vector. Total RNA was then collected and analyzed for *SNAI2*/Slug mRNA expression by Taqman Real-time PCR. shSlug decreased *SNAI2*/Slug mRNA expression by approximately 60%. shRNA-mediated knockdown of Slug protein was confirmed by Western blot (right panel). ***B***) Transwell migration assay for human U87 glioblastoma cells overexpressing an shRNA directed against *SNAI2*/Slug or a control vector. 10% serum was used as a chemoattractant. The cells that migrated through the membrane were stained with H&E and counted under direct microscopy. shSlug-expressing glioblastoma cells migrated slower than the control cells (asterisk indicates *P *< 0.0002, t-test). ***C***) 3-dimensional Matrigel assay for human U87 glioblastoma cells overexpressing an shRNA directed against Slug or a control vector. 24 hours after plating, cells that invaded through the matrix were stained and counted under direct microscopy. Data shown are mean ± SEM. Glioblastoma cells overexpressing shSlug showed less invasion than control cells (asterisk indicates *P *< 0.0001, t-test).

In order to determine the role of endogenous *SNAI2/*Slug in glioma invasion, we examined whether knockdown of *SNAI2*/Slug expression in glioblastoma cells with high endogenous expression of *SNAI2*/Slug could inhibit migration and invasion. The U87 human glioblastoma cell line was chosen for these studies because it displayed a high endogenous level of *SNAI2*/Slug mRNA and protein expression (cf. Figure 1D). We first generated lentiviruses containing either a control vector or an shRNA vector directed against *SNAI2*/Slug. Human U87 glioblastoma cells were then transduced with these lentiviruses to generate stable cell lines. Taqman Real-time PCR indicated that the *SNAI2*/Slug shRNA suppressed *SNAI2*/Slug mRNA expression by more than 60% when compared to control cells (Figure 5A). ShRNA-mediated knockdown of Slug protein was confirmed by Western blot (Figure 5A).

As shown in Figure 5B, U87 glioblastoma cells overexpressing *SNAI2*/Slug shRNA displayed decreased migration when compared to control cells (39 +/- 9 cells/hpf versus 72 +/- 7 cells/hpf, *P *< 0.0002, t-test), indicating a role for endogenous *SNAI2*/Slug expression in glioblastoma cell migration. To determine the effect of endogenous *SNAI2*/Slug on glioblastoma cell invasion, we again utilized the Matrigel invasion assay (Figure 5C). After *SNAI2*/Slug knockdown, significantly fewer U87 glioblastoma cells invaded through the matrix when compared to cells expressing an empty control vector (24 +/- 2 cells/hpf vs. 47 +/- 5 cells/hpf, *P *< 0.0001, t-test). Taken together, these findings indicate that endogenous *SNAI2*/Slug promotes the migration and invasiveness of human glioblastoma cells.

### SNAI2/Slug regulates glioma growth and angiogenesis in vivo

**Figure 6:**
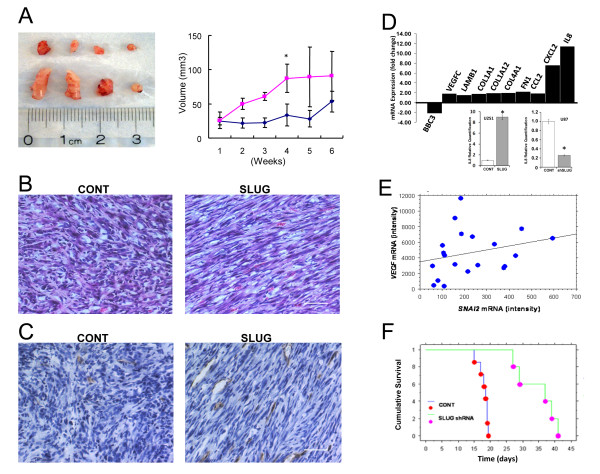
**Slug promotes glioblastoma growth and decreases survival in vivo**. ***A***) Human U251 glioblastoma cells transduced with a Slug-IRES-EGFP lentivirus or a control lentivirus were implanted subcutaneously into the flanks of nude mice. Quantitative data on tumor growth is shown on the right. Data shown are mean ± SEM (n = 4). Tumors overexpressing Slug grew at a faster rate than control tumors (*P *< 0.007 at 4 weeks, t-test). ***B***) Histological characteristics of U251 glioblastoma tumors shown in *A*). Sections are stained with hematoxylin and eosin. Note the spindle morphology of the Slug-overexpressing tumors. ***C***) CD31 immunoreactivity in U251 glioblastoma tumors overexpressing Slug or a control vector. Increased CD31 immunoreactivity was observed in Slug-overexpressing tumors, indicating the presence of increased vascularity (*P *< 0.002, t-test). ***D) **upper panel *mRNA microarray data obtained from human U251 glioblastoma cells transduced with either an *SNAI2 *lentivirus or a control virus. Data are expressed as fold change relative to mRNA expression in control cells. *lower panels *IL8 mRNA expression after Slug overexpression or Slug knockdown was validated by Real-time PCR in U251-IRES-Slug and U87-ShSlug glioblastma cells, respectively. Data shown are mean ± SEM of three replicates. ***E***) Regression plot of *SNAI2 *mRNA versus *VEGF *mRNA expression. mRNA microarray data was obtained from 20 human glioblastomas. *R*^2 ^= 0.068. ***F***) U87 human glioblastoma cells transduced with a Slug shRNA lentivirus or a control virus were transplanted into the brains of nude mice. Kaplan-Meier survival analysis indicated that *SNAI2/*Slug knockdown significantly improved survival (*P *< 0.0012, Logrank test).

The effects of Slug on glioma cell growth and invasion *in vitro *suggested that this transcription factor might promote glioma aggressiveness *in vivo*. We investigated this possibility using both subcutaneous and intracranial mouse transplantation models. First, we injected U251 glioblastoma cells transduced with a *SNAI2*/Slug lentivirus or a control lentivirus subcutaneously into the flanks of nude mice. Tumor size was then measured weekly for a period of 8 weeks. On average, human glioblastoma cells overexpressing *SNAI2*/Slug formed larger tumors than did control cells (Figure 6A, left panel). The average weight of tumors in which *SNAI2*/Slug was overexpressed was 101 +/- 43 mg, while that of control tumors was 38 +/- 10 mg. Growth curves indicated that glial tumors overexpressing *SNAI2*/Slug grew at a significantly faster rate *in vivo *than did control tumors (Figure 6A, *P *< 0.007 at 4 weeks, t-test).

The harvested subcutaneous tumors were also processed for histologic analysis. Tumor cells in which *SNAI2*/Slug was overexpressed displayed a more spindle-like morphology when compared to control cells (Figure 6B). Staining of tumor vasculature using a CD31 antibody (Figure 6C) revealed increased vascular proliferation in tumors in which *SNAI2*/Slug was overexpressed when compared to control tumors (19 +/- 4 CD31 positive vessels/hpf versus 8 +/- 1 CD31 positive vessels/hpf, *P *< 0.002, t-test). Thus, increased *SNAI2*/Slug expression promotes growth and angiogenesis in human gliomas, as has been reported for lung carcinoma [7].

Studies in other tissues indicate that *SNAI2*/Slug can increase the expression of angiogenic factors such as VEGF and basement membrane proteins[26], and can promote growth by repressing p53-mediated transactivation of the *BBC3*/PUMA gene[14]. We therefore examined whether *SNAI2*/Slug might have similar effects on gene expression in human glioma cells using mRNA microarray analysis. As observed in other cell types, overexpression of *SNAI2*/Slug in U251 glioblastoma cells decreased *BBC3*/PUMA mRNA expression and increased expression of angiogenic factors such as VEGF, IL8 and several extracellular matrix proteins by more than 1.5 fold (Figure 6D). To confirm that Slug increases IL8 expression, we quantified IL8 mRNA expression by Real-time PCR using U251-Slug-IRES-GFP and U87-sh-Slug glioblastoma cells. Overexpression of Slug increased IL8 mRNA expression approximately 10 fold, and knockdown of Slug in U87 cells significantly suppressed IL8 mRNA expression (Figure 6D).

Additional evidence for a correlation between *SNAI2*/Slug and VEGF was obtained using mRNA microarray data from primary glioblastoma samples. A plot of *VEGF *mRNA expression versus *SNAI2*/Slug mRNA expression in 20 human glioblastoma specimens revealed a weak but positive correlation (Figure 6E). This relationship was primarily due to the finding of low *VEGF *mRNA levels at very low levels of *SNAI2 *mRNA expression. Importantly, this same pattern was observed using an independent, publicly-available mRNA expression data set derived from approximately 50 human glioblastomas [27]. These findings suggest that, in addition to its effects on proliferation and invasion, *SNAI2*/Slug promotes angiogenesis in human glioblastomas.

To investigate whether knockdown of *SNAI2/*Slug could improve survival in a mouse intracranial human glioma transplantation model, we transplanted human U87 glioblastoma cells overexpressing a control vector or an shRNA vector directed against *SNAI2*/Slug into the brains of 5-week-old nude mice and followed the animals until they developed signs of distress due to glioblastoma growth. All mice with control U87 glioblastoma cell transplants died within 20 days, while mice transplanted with U87 glioblastoma cells overexpressing *SNAI2*/Slug shRNA survived as long as 42 days (Figure 6F). Kaplan-Meier analysis indicated that knockdown of endogenous *SNAI2/*Slug significantly prolonged survival (*P *< 0.0012, Logrank Test). Taken together, these data indicate that endogenous *SNAI2*/Slug promotes growth, invasion and aggressiveness in human glioblastomas.

## Discussion

Migration and invasion in cancer is a complex process requiring the coordinated action of numerous proteins and intracellular pathways. Evidence suggests that specific transcription factors coordinately regulate genetic programs promoting invasion and metastasis in a tumor-specific manner [2]. A characteristic feature of human malignant gliomas is their ability to diffusely invade normal brain tissues. Our findings demonstrate that aberrant expression of *SNAI2*/Slug, a master transcriptional regulator of invasion, contributes to this invasive behavior in gliomas. We observed that *SNAI2*/Slug expression correlates with histologic grade and tumor subtype, such that the most invasive gliomas (glioblastomas) displayed the highest levels of *SNAI2*/Slug expression. The degree of *SNAI2*/Slug overexpression in primary human glioblastoma specimens and its effects on growth and invasion by glioma cells are comparable to those of two other transcription factors involved in gliomagenesis, i.e. *HIF1A *and *STAT3*. Both overexpression and knockdown studies indicate that *SNAI2*/Slug increases glioblastoma growth and invasion *in vitro*. Moreover, inhibition of endogenous *SNAI2*/Slug expression improves survival in a mouse intracranial human glioblastoma transplantation model.

*SNAI2*/Slug acts as an oncogene in hematopoietic and other peripheral tissues [13]. *SNAI2*/Slug appears to have a function similar to that of the *TWIST1 *transcription factor, in that both can promote the epithelial-to-mensenchymal transition (EMT) in non-neural cells[9,22]. We observed that SNAI2/Slug overexpressing glioblastoma cells display a different phenotype and are more invasive than control glioblastoma cells, suggesting that they may have adopted more mesenchymal characteristics. Interestingly, a subclass of glioblastoma with increased mesenchymal differentiation has been reported[27]. Preliminary analysis in our laboratory indicates that *SNAI2 *mRNA is indeed upregulated in the mesenchymal subclass of glioblastomas (unpublished observations).

We observed that *SNAI2*/Slug increased the growth of both U251 and U87 human glioblastoma cells. In this respect, *SNAI2*/Slug differs from *TWIST1*, another transcription factor that promotes glioma invasion, which has no effect on glioma growth or proliferation[22]. The effects of *SNAI2*/Slug on glioma growth are likely related to the Slug-induced increase in DNA synthesis observed in glioblastoma cells in the current study. In addition, an anti-apoptotic effect of *SNAI2*/Slug has been reported [14]. In U87 glioblastoma cells, we observed a *SNAI2*/Slug-induced downregulation of mRNA expression for *BBC3*/PUMA, an effector of p53-induced apoptosis whose expression is transcriptionally repressed by *SNAI2*/Slug [14]. However, the finding that *SNAI2*/Slug promoted proliferation and tumor growth in U251 glioblastoma cells (which lack functional p53) suggests that mechanisms other than inhibition of the p53 pathway contribute to the effects of *SNAI2*/Slug on glioma growth.

## Conclusion

These data reveal a role for the invasion and metastasis-related transcription factor, *SNAI2*/Slug in human glioblastomas. We find that *SNAI2*/Slug increases the expression of *IL8*, and that *SNAI2*/Slug expression correlates positively with the expression of *VEGF *and other genes involved in glioma invasion and angiogenesis. In addition, we show that *SNAI2*/Slug activates a program of phenotypic changes in human glioblastoma cells that includes increased spindle morphology, vasculogenesis, growth and invasion. Importantly, *SNAI2*/Slug expression is increased by EGFR activation and promotes a mesenchymal phenotype in glioblastoma cells. Taken together, these findings reveal *SNAI2*/Slug to be an integral part of a growth factor-initiated genetic program promoting human glioblastoma growth and dispersal.

## Competing interests

The authors declare that they have no competing interests.

## Authors' contributions

HWY performed experiments and data analysis, participated in the study design and drafted the manuscript. LGM carried out immunohistochemistry and data analysis. PMB and RSC conceived of the study and participated in its design and coordination. MDJ developed the study design, performed experiments, participated in data analysis and interpretation and wrote the manuscript. All authors read and approved the final manuscript.

## Pre-publication history

The pre-publication history for this paper can be accessed here:

http://www.biomedcentral.com/1471-2407/10/301/prepub
